# Assessment of patient perception of glaucomatous visual field loss and its association with disease severity using Amsler grid

**DOI:** 10.1371/journal.pone.0184230

**Published:** 2017-09-26

**Authors:** Kenji Fujitani, Daniel Su, Mark P. Ghassibi, Joseph L. Simonson, Jeffrey M. Liebmann, Robert Ritch, Sung Chul Park

**Affiliations:** 1 Moise and Chella Safra Advanced Ocular Imaging Laboratory, Einhorn Clinical Research Center, New York Eye and Ear Infirmary of Mount Sinai, New York, NY, United States of America; 2 Bernard and Shirlee Brown Glaucoma Research Laboratory, Harkness Eye Institute, Columbia University Medical Center, New York, NY, United States of America; 3 Department of Ophthalmology, Manhattan Eye, Ear and Throat Hospital, New York, NY, United States of America; 4 Department of Ophthalmology, Zucker School of Medicine at Hofstra/Northwell, Hempstead, NY, United States of America; The University of Melbourne, AUSTRALIA

## Abstract

**Purpose:**

To investigate patients’ perception of glaucomatous VF loss and its association with glaucoma severity using the Amsler grid test.

**Methods:**

In this prospective cross-sectional study, glaucoma patients with abnormal 10–2 Humphrey Swedish Interactive Threshold Algorithm-standard VF tests were enrolled consecutively. All patients underwent a black-on-white Amsler grid test for each eligible eye. They were asked to outline any perceived scotomas (areas with abnormal grid lines) on the grid and then describe verbally their perception of the scotomas. Examiners asked patients to clarify their descriptions. All descriptions used by patients were recorded in their own words, which were then sorted into descriptor categories according to similar themes. The number of descriptor categories was counted for each eye. 10–2 VF mean deviation (MD) was compared among eyes that reported different number of descriptor categories. The mean 10–2 VF MD values were compared among different descriptor categories.

**Results:**

Fifty glaucoma patients (88 eyes) were included. Patients used a total of 44 different descriptors for their scotomas. Patients’ descriptors were classified into categories that incorporated similar themes, resulting in 4 overarching descriptor categories: Missing/White, Blurry/Gray, Black, and Not Aware. Fifty-two eyes reported one descriptor category and 19 eyes reported two descriptor categories (mean number of descriptor categories = 1.27±0.45). Eyes that reported two descriptor categories had worse VF MD than those that reported one (-17.86±10.31 dB vs. -12.08±7.53 dB; p = 0.012). When eyes were organized according to its combination of descriptor categories, each eye naturally sorted into one of the following 5 groups, in frequency order: Missing/White (27 eyes; 31%), Blurry/Gray (21 eyes; 24%), combined Missing/White and Blurry/Gray (19 eyes; 21%), Not Aware (17 eyes; 19%), and Black (4 eyes; 5%). The mean 10–2 VF MD severity order was Black (-21.18±10.59 dB), combined Missing/White and Blurry/Gray (-17.86±10.31 dB), Missing/White (-11.92±6.76 dB), Blurry/Gray (-10.55±7.03 dB), and Not Aware (-3.91±4.05 dB) (p<0.001).

**Conclusion:**

Paracentral vision loss in glaucoma is perceived by patients. As the perception of scotomas and the variety of terms to describe scotomas are related to glaucoma severity, clinicians should pay attention to patients’ subjective descriptions of their glaucomatous VF loss. The historical notion that glaucoma patients lose their peripheral vision first and eventually look through a black tunnel needs to be updated to reflect the true perception of glaucoma.

## Introduction

Glaucoma is the second leading cause of blindness worldwide[1] and is estimated to affect 80 million people by the year 2020 [2]. While automated perimetry is widely used in research and clinical practice as the main measure of glaucoma patients’ visual deficits [3–5], much is unknown about what they actually see [6]. The NIH website [7] describes that at first, open-angle glaucoma has normal vision with no symptoms but with glaucoma progression, patients lose their vision over time. It continues that people with glaucoma slowly lose their peripheral vision and eventually seem to be looking through a tunnel [7]. However, with advancements in understanding glaucoma, the description of glaucomatous visual loss as black tunnel vision has come into question.

Crabb et al [8] found that glaucoma patients frequently opted for terms such as “missing” and “blur” rather than “black” to describe their visual field (VF) loss. Some patients were unaware of their VF loss [8]. Hu et al [9] reported that most of the time, vision loss in glaucoma was characterized by blurry vision and needing more light. Accurate understanding of patients’ perception of glaucomatous VF loss and how it affects them in their daily lives are helpful to diagnose, monitor, and increase adherence to treatment for glaucoma [10]. An improved understanding of the relationship between patient-reported visual dysfunction and glaucoma severity is also helpful to glaucoma management.

We have described the utility of the Amsler grid in glaucoma and found that it could identify VF defects within the central 10 degrees with an overall specificity of 92% and positive predictive value of 97% [11]. In the present study, we used the Amsler grid to better understand patients’ perception of glaucomatous visual loss and sought to describe the association between patients’ perception of scotomas and glaucoma severity.

## Materials and methods

This prospective, cross-sectional study was approved by the Institutional Review Board for Human Research of the New York Eye and Ear Infirmary. Written informed consent was obtained from all subjects and the study adhered to Health Insurance Portability and Accountability Act and the tenets of the Declaration of Helsinki.

Consecutive glaucoma patients with a range of optic disc and VF abnormalities representing various stages of glaucomatous damage were prospectively recruited from August 1, 2011 to December 31, 2011. Glaucoma was defined by the presence of characteristic glaucomatous optic disc and/or retina changes (localized or diffuse neuroretinal rim thinning or retinal nerve fiber layer defect) associated with corresponding reproducible VF defects on 24–2 Swedish Interactive Threshold Algorithm (SITA) standard VF test (Humphrey Field Analyzer II; Carl Zeiss Meditec, Inc., Dublin, CA). A glaucomatous VF defect was defined as a glaucoma hemifield test result outside normal limits on two consecutive VF tests and the presence of at least 3 contiguous test points within the same hemifield on pattern deviation plot at p<0.01, with at least 1 point at p<0.005. These tests required reliability indices better than 15%.

Among these patients, those with abnormal 10–2 SITA standard VF test (Humphrey Field Analyzer II) on the date of enrollment or within the previous 3 months were enrolled for the Amsler grid test. An abnormal 10–2 VF was defined as the presence of at least 3 contiguous test points within the same hemifield on the pattern deviation plot at p<0.01, with at least 1 point at p<0.005. The VF tests required reliability indices better than 15%. Patients were excluded from the study if they had ocular or systemic conditions other than glaucoma known to affect the VF, inability to perform reliable perimetry, posterior segment intraocular surgery, or any ocular surgery between the 10–2 VF and Amsler grid tests.

Baseline demographic characteristics were recorded and the Amsler grid test was administered for the right eye and then the left eye of enrolled subjects. The procedure for administering the Amsler grid tests was identical to that used in our previous study [11]. After correcting for near refractive error, a black-on-white Amsler grid test was administered for each eligible eye at a distance of 30 cm, the distance at which each box on the grid corresponds to 1 degree of VF [12]. The same examination room with the same lighting conditions was used for all patients to standardize testing conditions. An eye patch occluded the eye not being tested, and the patients were instructed to fixate on the central point of the grid at all times. For those patients who had difficulty of fixation due to a moderate to severe central VF defect, a grid with two diagonal lines connecting the corners of the grid was utilized to assist in extrapolating the central location. Patients were asked to outline any perceived scotomas (areas with abnormal grid lines) on the grid with a pencil and then describe verbally their perception of the scotomas in detail. Each patient was asked the open-ended question: “please describe what you see.” Examiners asked patients to clarify their descriptions. All descriptions used by patients were recorded in their own words, which were then sorted into descriptor categories according to similar themes.

The number of descriptor categories was counted for each eye. If an eye used multiple descriptors that fit in the same descriptor category, it was considered to have mentioned only 1 category. Next, 10–2 VF mean deviation (MD) was compared among eyes that reported different number of descriptor categories, using analysis of variance with post-hoc (Least Significant Difference) or independent t-test. Finally, mean 10–2 VF MD values were compared among different descriptor categories using analysis of variance with post-hoc (Least Significant Difference). Microsoft Office Excel for Windows (Microsoft, Redmond, WA) and SPSS version 20.0 for Windows (SPSS Inc., Chicago, IL) were used for statistical analysis. Tukey’s hinges percentiles were used for box-and-whisker plots. A p value <0.05 (two-tailed) was considered significant.

## Results

A total of 50 patients with abnormal 10–2 SITA standard VF test on the date of enrollment or within the previous 3 months were approached and all of them agreed to participate in the Amsler grid test. We included 88 eyes of 50 glaucoma patients (mean 10–2 VF MD, -11.75±8.86 dB; mean age, 67±11 [range, 34 to 89] years; 17 males and 33 females). The remaining 12 eyes had no glaucomatous VF loss on 10–2 SITA standard VF test. It took approximately 3 minutes to perform the Amsler grid test on one eye including outlining perceived scotomas and describing verbally their perception of the scotomas. Patients’ perception of glaucomatous VF loss within the central 10 degrees varied considerably. Patients used a total of 44 different descriptors to explain their perception of scotomas. After reviewing each patient’s descriptions of his or her perceived scotomas, we classified 40 of the 44 descriptors into categories that incorporated similar themes together. This resulted in 6 overarching categories: Blurry, Gray, Missing, White, Black, and Not Aware (**Table 1**). Some categories contained more descriptors than others. The remaining 4 descriptors did not fit in the above 6 categories: ‘areas with ripples like raindrops in a puddle,’ ‘clear areas looks like a flashlight,’ ‘something funny or wrong,’ and ‘looks like lines are shiny and reflecting light.’ These 4 descriptors were used by 4 different subjects, and were always reported alongside one or more of the 40 other descriptors in **Table 1**. We believed that these 4 descriptors may be supplementary expansions of the 40 descriptors in **Table 1**, so they were not used in the analysis.

**Table 1:** Categorization of verbal responses used by glaucoma patients to describe their visual field loss.

4 Descriptor Categories	6 Descriptor Categories	Words/Phrases Used by Patients to Describe Visual Field Loss (Descriptors)
Blurry/Gray	Blurry	blurry, general blurriness, hazy, very hazy, foggy, cloud covering, smog, faded, watery, less clear, less crisp, not as crisp, not as sharp, fuzzier than surrounding, not as defined, opaque glass with some light visible
	Gray	gray, gray-out, light gray, gray shadow, shadow
Missing/White	Missing	missing, missing areas, just missing, almost missing, disappears sometimes, washed-out, not much there, lines are gone, “I know it is there but I cannot see it”
	White	white, white-out, a little white, white area, bone white
Black	Black	more black, black-out, big black blur, dark
Not Aware	Not Aware	not aware

**Fig 1:**
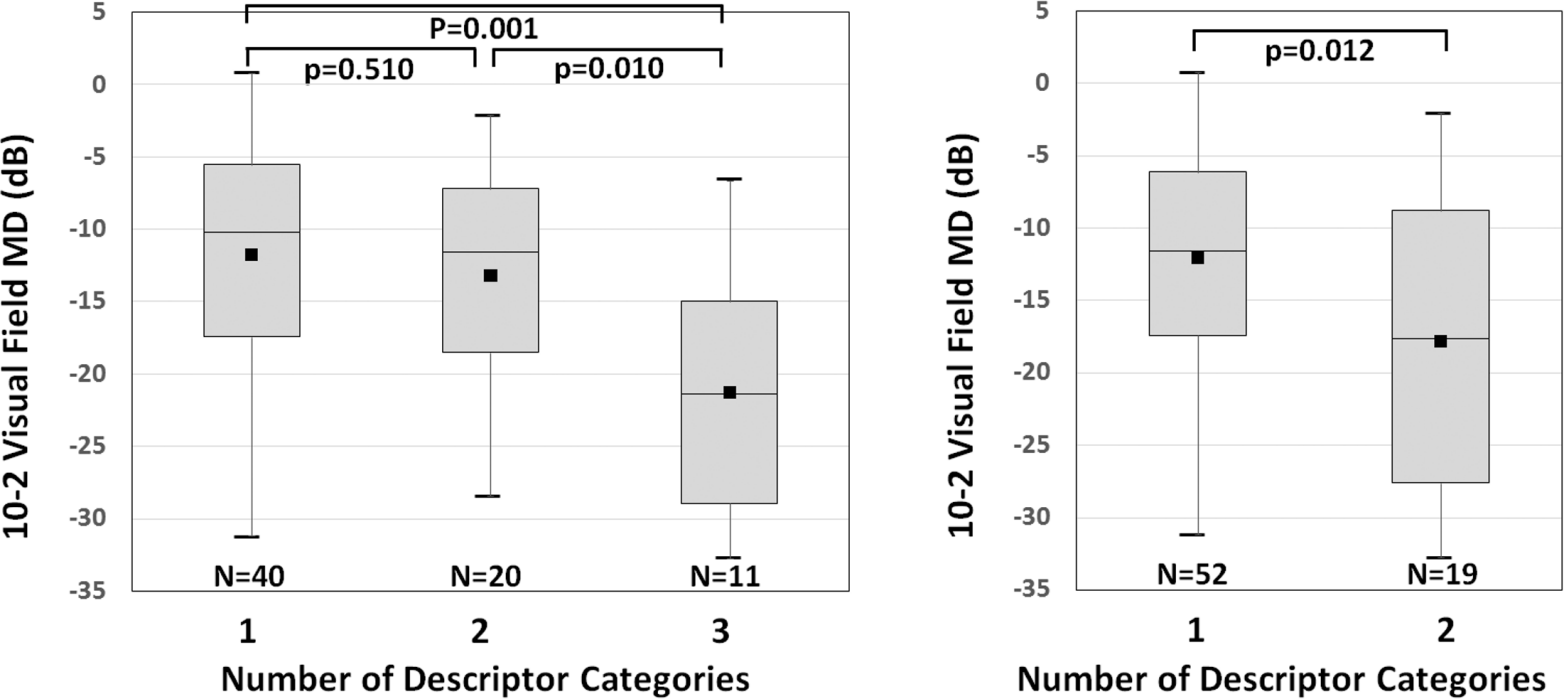
Box-and-whisker plots of 10–2 visual field mean deviation (MD) according to the number of descriptor categories per eye (a total of 71 eyes with Amsler grid scotomas). (**Left**) For 6 descriptor category classification in **Table 1**, and (**Right**) for 4 descriptor category classification in **Table 1**. The horizontal line within the box indicates the median, upper and lower boundaries of the box indicate the 75th and 25th percentiles, respectively, and the whiskers indicate the maximum and minimum values. The black squares in the boxes indicate the mean. Asterisks indicate p values <0.05.

Amsler grid scotomas (areas with abnormal grid lines) were noted in 71 of 88 eyes (81%) but absent in 17 eyes (19%; ‘Not Aware’ category). For each of the 71 eyes with Amsler grid scotomas, one to three descriptor categories were used to describe the perception of its scotomas and the mean number of descriptor categories per eye was 1.59±0.75 (range, 1–3). Eyes with greater number of descriptor categories had worse VF MD (10–2 VF MD = -21.26±9.37 dB in eyes with three descriptor categories, -13.21±8.06 dB in eyes with two descriptor categories, and -11.74±7.80 dB in eyes with one descriptor category; p = 0.004 by analysis of variance; **Fig 1A**).

**Fig 2:**
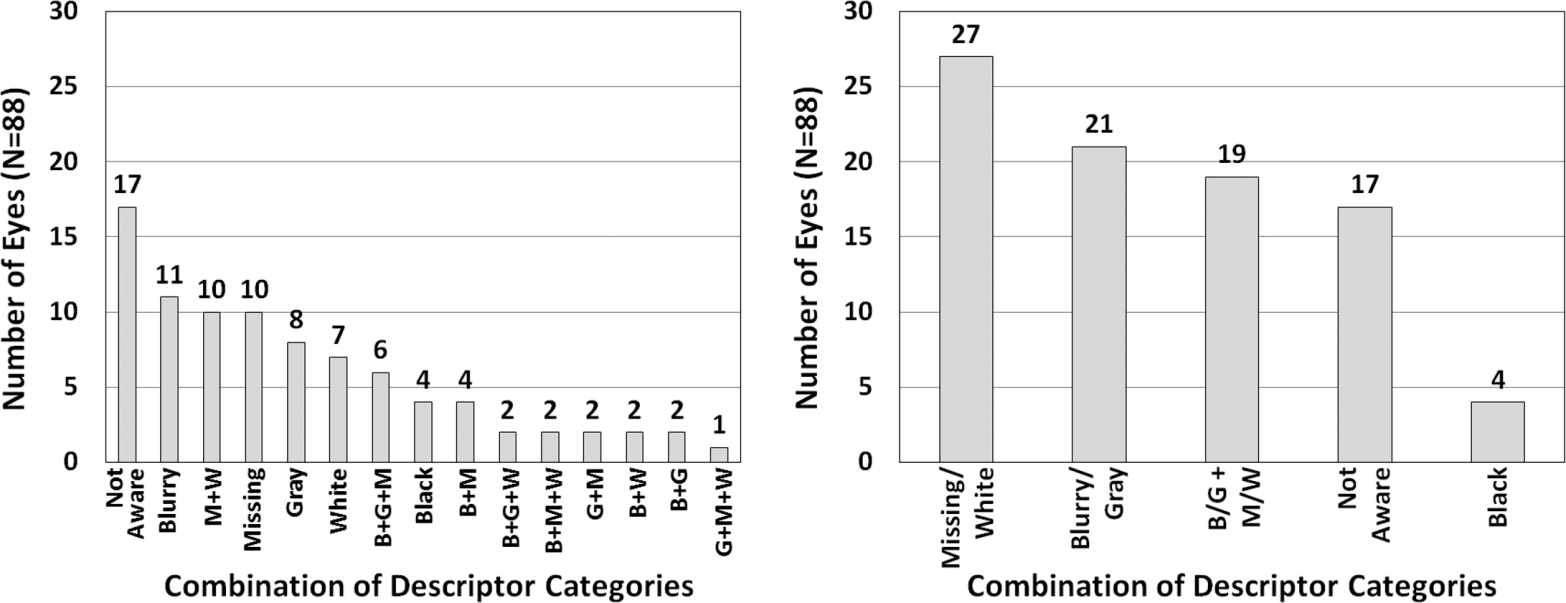
Number of eyes for each combination of descriptor categories. The number above the bar graphs represents the number of eyes in that combination (a total of 88 eyes). (**Left**) For 6 descriptor category classification in **Table 1**, and (**Right**) for 4 descriptor category classification in **Table 1**. A ‘+’ mark represents a combination of descriptor categories. B = Blurry, and G = Gray, M = Missing, W = White.

In order to analyze which perceptions were more prevalent than others and to determine how they related to glaucoma severity, eyes were organized into groups according to its combination of descriptor categories. For instance, an eye with one descriptor in the ‘Blurry’ category and another descriptor in the ‘Missing’ category would be counted as ‘Blurry+Missing’ to signal a combination of ‘Blurry’ and ‘Missing’ categories. This eye would not be counted in either ‘Blurry’ or ‘Missing’ category. Based on the 6 descriptor categories in **Table 1**, each eye naturally sorted into one of the following 15 groups, in frequency order: Not Aware (17 eyes, 19%), Blurry (11 eyes, 13%), Missing+White (10 eyes, 11%), Missing (10 eyes, 11%), Gray (8 eyes, 9%), White (7 eyes, 8%), Blurry+Gray+Missing (6 eyes, 7%), Black (4 eyes, 5%), Blurry+Missing (4 eyes, 5%), Blurry+Gray+White (2 eyes, 2%), Blurry+Missing+White (2 eyes, 2%), Gray+Missing (2 eyes, 2%), Blurry+White (2 eyes, 2%), Blurry+Gray (2 eyes, 2%), and Gray+Missing+White (1 eye, 1%) (**Fig 2A**).

However, with 15 different groups, analyzing and interpreting the data with regard to glaucoma severity (VF MD) was complex and did not appear to be clinically helpful. Therefore, we merged descriptor categories that could be combined based on their similarities. For instance, because the Amsler grid was administered on a white background, ‘Missing’ and ‘White’ could represent the same phenomena, and thus combined to generate a new category, ‘Missing/White.’ Likewise, when black grid lines on the Amsler grid become blurry, they may appear gray, so ‘Blurry’ and ‘Gray’ were consolidated into the same category ‘Blurry/Gray.’ In consequence, the aforementioned 6 descriptor categories were consolidated into 4 new categories: Missing/White, Blurry/Gray, Black, and Not Aware (**Table 1**).

In the 71 eyes with Amsler grid scotomas (excluding the 17 eyes in the ‘Not Aware’ category), the mean number of new descriptor categories per eye was 1.27±0.45 (range, 1–2). Eyes that reported two descriptor categories had worse VF MD than those that reported one (10–2 VF MD = -17.86±10.31 dB vs. -12.08±7.53 dB; p = 0.012 by independent t-test; **Fig 1B**). When eyes were organized into groups according to their combination of new descriptor categories, each eye naturally sorted into one of the following 5 groups, in frequency order: Missing/White (27 eyes; 31%), Blurry/Gray (21 eyes; 24%), combined Missing/White and Blurry/Gray (19 eyes; 21%), Not Aware (17 eyes; 19%), and Black (4 eyes; 5%) (**Fig 2B**).

**Fig 3:**
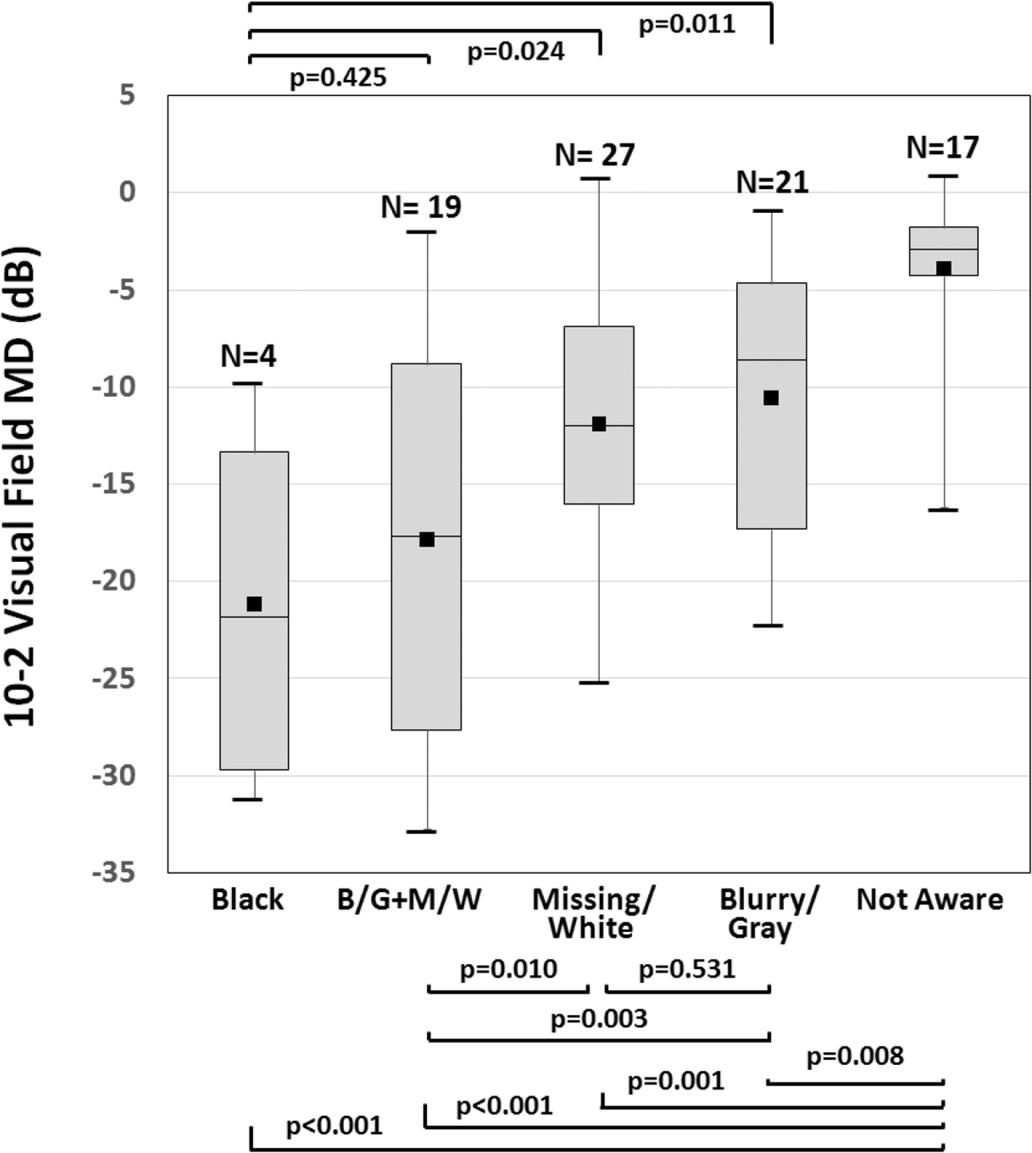
Box-and-whisker plots of 10–2 visual field mean deviation (MD) according to the 5 combinations of descriptor categories of the eye (4 descriptor category classification in Table 1). The number above the bar graphs represents the number of eyes in that combination (a total of 88 eyes). The horizontal line within the box indicates the median, upper and lower boundaries of the box indicate the 75th and 25th percentiles, and the whiskers indicate the maximum and minimum values. The black squares in the boxes indicate the mean. ‘B/G+M/W’ indicates combination of ‘Blurry/Gray’ and ‘Missing/White’ descriptor categories. Overall p value <0.001. Asterisks indicate p values <0.05.

Mean 10–2 VF MD values among the 5 groups were significantly different (p<0.001 by analysis of variance, **Fig 3**). The 10–2 VF MD severity order was Black (-21.18±10.59 dB), combined Missing/White and Blurry/Gray (-17.86±10.31 dB), Missing/White (-11.92±6.76 dB), Blurry/Gray (-10.55±7.03 dB), and Not Aware (-3.91±4.05 dB). The ‘Not Aware’ group had significantly better 10–2 VF MD than the other groups (all p<0.009, **Fig 3**). The ‘Black’ group had significantly worse 10–2 VF MD than the other groups (all p<0.025), except that the difference between the ‘Black’ group and the ‘combined Missing/White and Blurry/Gray’ group was not statistically significant (p = 0.425) (**Fig 3**).

## Discussion

In our previous study [11], we showed that the Amsler grid could detect glaucomatous central VF loss with high specificity (92%) and high positive predictive value (97%). The test’s sensitivity increased as glaucoma advanced: 40% in eyes with 10–2 VF MD better than -6 dB, 58% in eyes with 10–2 VF MD between -12 and -6 dB, and 92% in eyes with 10–2 VF MD worse than -12 dB [11]. This suggests that the Amsler grid may be a simple yet useful tool for evaluating central VF loss in glaucoma. Using the Amsler grid, the present study shed light on how patients perceive their glaucomatous VF loss. We demonstrated that patients’ perception of glaucomatous VF loss within the central 10 degrees varied considerably (**Table 1**), and that patients who used a greater variety of terms to describe their scotomas had more advanced glaucoma (**Fig 1**). Patients frequently opted for descriptors such as blurry and missing (**Fig 2B**). Approximately 1 of 5 eyes did not recognize their scotomas and approximately 1 of 20 eyes perceived scotomas as black or dark areas (**Fig 2B**). In general, patients unaware of their glaucomatous VF defects had milder glaucoma than others, and those perceiving black or dark scotomas had more advanced glaucoma than others (**Fig 3**).

Similar studies have evaluated patients’ perception of glaucomatous VF loss. Crabb et al [8] recorded interviews and placed patients in a forced-choice experiment in which patients chose one figure that best represented their glaucoma perception among 6 predetermined figures. They found glaucoma patients did not perceive their vision loss as either “black patches” or “black tunnels,” but rather found 54% of patients with “blurred parts,” 26% with “not aware,” and 16% with “missing parts.” Hu et al [9] administered one questionnaire twice with 25 yes or no visual symptom questions and 3 open ended questions about vision changes over time. They found that 58% of early or moderate glaucoma patients reported needing more light, 52% had blurry vision, and 52% had glare. Patients with more severe glaucoma were more likely to report difficulty seeing objects to the side, as if looking through dirty glasses, and trouble differentiating colors and boundaries.

Unlike previous studies investigating glaucoma patients’ perception [8,9], ours used the Amsler grid as a reference tool, placed no restrictions, and allowed patients to individually depict their scotomas according to how they were perceived. The patients verbally explained their perception of scotomas as they marked the scotomas on the Amsler grid. That is, while the previous studies [8,9] were based on patients’ recall of their perception of scotomas, we asked for real-time perceptions.

There are advantages to both monocular and binocular studies. The previous studies [8,9] used binocular perception, which may mask glaucomatous VF loss in one eye when the other eye is compensating. Because patients use both eyes in their real life, however, the results of binocular studies can easily be generalized. In the current study, patients’ perceptions were obtained from each eye to avoid the confounding effect of the fellow eye. However, our results may be less able to be generalized into real life than the results of binocular studies.

There was a trend for eyes with more descriptor categories to have worse VF MD. That is, diverse perception problems generally meant more advanced glaucoma. This suggests that an addition of a new descriptor category could signal glaucoma progression. Therefore, when glaucoma patients are explaining their VF loss, it may be important for clinicians to notice whether they use synonyms of an already mentioned descriptor or a new descriptor that fits into a different category.

Classic glaucoma perception has long been described as a black tunnel or patch [7], but Crabb et al [8] found that none of the patients chose the two black images in a forced choice experiment and Hu et al [9] found no patient to report tunnel vision. As reported in these previous studies [8,9], we found Missing/White and/or Blurry/Gray to be the predominating perception of glaucomatous VF defects. Therefore, the current depiction of glaucoma as explained in government and mass public information documents and websites such as the NIH [7] needs to be updated to reflect the true perception of glaucoma. In our study, 4 of 88 eyes (5%) recognized their scotomas as black or dark. We believe that perception of black still exists in some patients with advanced glaucoma, albeit uncommonly. The most likely explanation for different results regarding the presence of black perception between our study and the previous study [8] may lie in methodology. In the previous study [8], glaucoma patients were told to choose one of six images that best represented their perception of VF loss. Two of the six images contained black areas but patients’ black perception may not have been depicted accurately by the two images. The restriction-free methodology using the Amsler grid in our study may have enabled patients to recognize their black perception.

Consistent with previous studies [8,9], patients who were unaware of their VF loss had significantly better 10–2 VF MD values than others. This is probably because patients do not recognize early glaucomatous VF defects since the brain fills in the defects [6]. Conventional concept of glaucomatous VF defects hypothesized that patients develop peripheral VF defects first, which then progress to more central VF defects. This initial development of VF defects in the periphery has been considered as the reason for patients’ inability to recognize visual disturbances or VF defects until glaucoma advances to later stages. However, based on our results, glaucomatous VF defects may not be recognized in the early stages of the disease even if they are present within the central 10 degrees.

Based on **Fig 3**, 10–2 VF MD tended to worsen in the following order: Not Aware, Blurry/Gray, Missing/White, combined Blurry/Gray and Missing/White, and Black. Intuitively, loss of retinal ganglion cells in glaucoma leads to loss of visual perception. Initially, the loss of visual perception may not be sufficient for the patient to detect, leading to unawareness. With increasing loss of retinal ganglion cells, the decrease in visual signals reaching brain can be recognized as Blurry/Gray perception. More loss of retinal ganglion cells may cause deepening of pre-existing scotomas and create a Missing/White perception. More extensive glaucoma progression may lead to a combination of Missing/White and Blurry/Gray perception as the increasing loss of retinal ganglion cells affects a larger area and becomes more concentrated in some localized areas. In some patients, severe loss of retinal ganglion cells could be recognized as black or dark, but its mechanism is unclear.

This study is not without limitations. First, the classification of patients’ verbal responses is subjective, so one could argue that the descriptors could be differentiated into different categories besides the one we presented. Second, the 4 descriptors that did not fit in the 6 categories in **Table 1**were excluded from analysis (‘areas with ripples like raindrops in a puddle,’ ‘clear areas looks like a flashlight,’ ‘something funny or wrong,’ and ‘looks like lines are shiny and reflecting light’). We believed that these 4 descriptors may be supplementary expansions of the 40 descriptors in **Table 1**, but we cannot rule out the possibility of another descriptor category that only a small subset of glaucoma patients perceive. Third, our results depend on the use of Amsler grid. The glaucoma severity tendency of Black > ‘combined Missing/White and Blurry/Gray’ > Missing/White > Blurry/Gray > Not Aware may not change, but the proportion of eyes in each descriptor category is subject to change depending on the type of test administered. Fourth, the Amsler grid test was administered for the right eye and then the left eye of enrolled subjects. The responses for the first eye tested could influence those for the second eye, which could introduce a bias. Finally, part of our results obtained from the two eyes of the same patient may be correlated, because a patient may use similar descriptions for his or her VF defects in both eyes. However, it was difficult to perform a statistical adjustment to account for this correlation appropriately because most patients have a different severity of VF defects in one eye from the other. It should be noted that our p values are likely overestimated due to this correlation [13].

In conclusion, patient perception of glaucomatous VF loss within the central 10 degrees varied considerably. Based on our results, predominant perception of glaucoma patients is Blurry/Gray and/or Missing/White areas, differing from the traditional concept. This result will be helpful for clinicians to explain whether patients’ visual symptoms are related to glaucoma or other conditions. Patients who used a greater variety of terms to describe their scotomas had more advanced glaucoma, and patients’ perception of scotomas was related to glaucoma severity. Healthcare providers should explain glaucoma perception correctly, so that patients can be more aware of what to expect for development or progression of glaucoma. When glaucoma patients are explaining their VF defects, it is important for clinicians to notice whether they are saying synonyms of an already mentioned descriptor or a new descriptor that fits into a different category. Amsler grid test can provide patients an opportunity to understand the impact of their glaucomatous VF loss, and this may improve their adherence to medications. Future research could determine if any associations exist between patient perception and VF defect extent, depth, or location.

## Supporting information

S1 File Data underlying the findings described in the manuscript. (XLSX)
